# Higher Parenteral Electrolyte Intakes in Preterm Infants During First Week of Life: Effects on Electrolyte Imbalances

**DOI:** 10.1097/MPG.0000000000003532

**Published:** 2022-06-20

**Authors:** Cornelia Späth, Elisabeth Stoltz Sjöström, Magnus Domellöf

**Affiliations:** From the * Department of Clinical Sciences, Pediatrics, Umeå University, Umeå, Sweden; the † Department of Food, Nutrition and Culinary Science, Umeå University, Umeå, Sweden.

**Keywords:** chloride, hypernatremia, hypokalemia, potassium, preterm infants, sodium

## Abstract

This study aimed to investigate the effects of a higher intake of electrolytes from parenteral nutrition (PN) on plasma electrolyte concentrations in very low birth weight (VLBW, <1500 g) infants.

**Methods:**

This was a single-center cohort study including all VLBW infants born before (n = 81) and after (n = 53) the implementation of a concentrated PN regimen. Daily nutritional intakes and plasma concentrations of sodium, chloride, potassium, phosphate, and calcium were collected from clinical charts.

**Results:**

During the first postnatal week, electrolyte intakes were higher in infants who received concentrated PN compared with infants who received original PN. Infants who received concentrated PN had a lower incidence of hypokalemia (<3.5 mmol/L; 30% vs 76%, *P* < 0.001) and severe hypophosphatemia (<1.0 mmol/L; 2.2% vs 17%, *P* = 0.02). While the relatively high prevalence of severe hypophosphatemia in infants who received original PN can be explained by a phosphorus intake below the recommendation, the potassium intake during the first 3 postnatal days (mean ± SD: 0.7 ± 0.2 mmol/kg/d) was within the recommendation. The prevalence of early hypernatremia was not affected by the different sodium intake in the 2 groups.

**Conclusions:**

In VLBW infants, a sodium-containing PN solution (about 2.7 mmol/100 mL) does not cause hypernatremia during the first days of life. Furthermore, providing at least 1 mmol potassium/kg/d during the first 3 postnatal days might be necessary to prevent early hypokalemia.

**What Is Known**

- Electrolyte imbalances are common during the first postnatal days in very low birth weight infants.
- Sodium and potassium intakes from parenteral nutrition (PN) are recommended to be minimized during the first 3 postnatal days (0–2 and 0–3 mmol/kg/d, respectively).

**What Is New**

- A PN solution with higher electrolyte concentrations resulted in intakes closer to the recommendations and less electrolyte imbalances.
- A sodium intake of 2.0 mmol/kg/d from PN during the first 3 postnatal days did not result in increased incidence of hypernatremia.
- A minimum potassium intake of about 1 mmol/kg/d from PN during the first 3 days was found to prevent early hypokalemia.

Parenteral nutrition (PN), using standardized ready-to-use high-density solutions, is recommended from the day of birth for very low birth weight (VLBW, <1500 g) infants to ensure an anabolic state as soon as possible (1). The physiological postnatal adaptation as well as the composition of the PN solution have considerable effects on electrolyte homeostasis. This is especially relevant since abnormalities in plasma concentrations of sodium, potassium, phosphate, and calcium are commonly observed in preterm infants during the early postnatal period (2–5).

To meet the recommended PN intakes in preterm infants, a concentrated PN regimen was implemented at the neonatal intensive care unit at Umeå University Hospital, Umeå, Sweden (6). We have previously shown that VLBW infants who received the concentrated PN regimen had increased early energy and protein intakes and showed improved postnatal growth compared with infants who received the original PN regimen (7). However, there is currently a lack of evidence concerning the optimal concentrations of electrolytes in PN solutions for use during the first days of life in VLBW infants. In this present study the aim was to investigate how the change to the concentrated PN regimen affected (1) intakes and (2) plasma concentrations of sodium, chloride, potassium, phosphate, and calcium during the first postnatal week.

## METHODS

### Study Design and Patients

In this single-center retrospective observational cohort study, all VLBW infants born between February 1, 2010, and September 30, 2013, admitted to the neonatal intensive care unit at Umeå University Hospital (Umeå, Sweden) within 24 hours after birth, and treated there for at least 7 days were included (n = 134). We excluded 7 infants because of chromosomal or severe congenital anomalies, one infant because of a complete lack of nutritional records and another infant because of not receiving the studied PN regimen during the first postnatal week. On February 19, 2012, a concentrated PN regimen was introduced at the neonatal intensive care unit resulting in group sizes of 79 and 46 infants who received the original PN and the concentrated PN regimen, respectively. The smaller group size in the latter group was due to an alteration of the PN protocol due to a temporary lack of availability of the concentrated PN solution.

### Parenteral Nutrition

During the total study period, for the vast majority of infants, PN was administered using a central venous line. The original PN regimen consisted of a pharmacy-prepared all-in-one bag combined with a glucose bag containing individually added electrolytes. The concentrated PN regimen consisted of a commercially available bag containing glucose, amino acids, and electrolytes (Numeta G13, Baxter Medical AB, Stockholm, Sweden) with a routine addition of trace elements and the possibility to add further electrolytes. This bag was combined with a lipid bag based on soybean oil, medium chain triglycerides, olive oil, and fish oil (SMOF, Fresenius Medical Care AG & Co. KGaA, Bad Homburg, Germany) with added fat- and water-soluble vitamins. The electrolyte and macronutrient contents of the parenteral bags are shown in Table, Supplemental Digital Content 1, http://links.lww.com/MPG/C860. Regardless of the PN regimen, some infants received a further adjustable glucose, lipid, and amino acid bag containing electrolytes. Besides the introduction of the concentrated PN regimen, no other changes regarding PN routines were made during the study period.

### Enteral Nutrition

Enteral nutrition was started as soon as possible after birth and increased according to clinical decision for all infants. With a few exceptions, enteral nutrition consisted exclusively of mother’s own breast milk or donor breast milk. A human milk fortifier (Nutriprem, Nutricia Nordica AB, Stockholm, Sweden) was routinely added to breast milk starting at 70 to 100 mL/kg/d enteral fluid volume and individually adjusted according to breast milk macronutrient analysis (Milkoscan 4000, FOSS, Hillerød, Denmark).

### Data Collection and Management

Daily nutritional intakes were retrospectively collected from clinical charts from the day of birth (day 0) to day 6 and presented during days 0 to 3 and 4 to 6 to facilitate a comparison with the current PN guidelines (8,9). All nutrient intakes were calculated between 6 am and 6 am the following day. Therefore, nutrient intakes of postnatal day 6 ended in the morning of postnatal day 7. Nutrient intakes on the day of birth were corrected for time of birth. Electrolyte supply from nutritional products including saline injections/infusions and transfused blood products was calculated using manufacturer-supplied information and published nutrient contents (10,11). Enteral calcium intakes were also adjusted for its low estimated intestinal absorption rate of 50.3% (12).

The first available measurements of pH, base excess, and plasma electrolyte concentrations each day were retrospectively collected from clinical charts for postnatal days 0 to 7. Plasma concentrations of sodium, chloride, potassium, and ionized calcium were analyzed with a point-of-care blood gas analyzer (ABL 800, Radiometer, Brønshøj, Denmark) in daily clinical routine. Blood samples for phosphate were obtained on average 1.8 times in infants who received the original PN regimen and 3.9 times in infants who received the concentrated PN regimen. All plasma phosphate concentrations were analyzed in clinical chemistry laboratory. Electrolyte imbalances were defined in accordance with the scientific literature (4,8,13–15). Reference intervals for chloride are not well defined in preterm infants. We used the ±2 SD intervals from Iacobelli et al (16), providing a range of 100 to 120 mmol/L during the first week of life.

Perinatal and clinical outcome data were defined in accordance to the literature (17–19) and collected from the Swedish Neonatal Quality Register (www.snq.se) except for data on retinopathy of prematurity (ROP) that were collected within the Swedish National Register for ROP. Birth weight was transformed into standard deviation score for gestational age according to Niklasson and Albertsson-Wikland (20) and has previously been described (7).

### Statistical Analyses

A post hoc power analysis showed that with group sizes of 79 and 46 infants and a significance level of 0.05, we achieved a power of 0.76 to detect a difference in electrolyte concentrations of medium effect size (Cohen d = 0.5) (21). Data were analyzed using SPSS (IBM, SPSS Version 25.0 for Windows, Armonk, NY). To evaluate differences between the groups, we used the Independent samples *t* test and the Fisher exact test for continuous and binary outcome variables, respectively. For those clinical outcome variables which were not normally distributed (Apgar score at 5 minutes, days of mechanical ventilation treatment, and days of antibiotics treatment), Mann-Whitney *U* test was used. To control for the different multiple pregnancy rate, respective multivariable regression analyses were used. Since the number of missing data entries was very low, listwise deletion in each analysis was used for missing data.

### Ethical Approval Information

The study was approved by the Regional Ethical Review Board at Umeå University in Umeå, Sweden (Dnr 2011-417-31 M, 2012-458-31 M, and 2017-35-32 M). The Ethical Review Board waived the requirement for informed consent in this retrospective study.

ClinicalTrials.gov Identifier: NCT04085484, https://clinicaltrials.gov/ct2/show/NCT04085484?term=PUMPA&draw=2&rank=1.

## RESULTS

There were no differences in perinatal characteristics between infants who received the original PN regimen (original PN group) and infants who received the concentrated PN regimen (concentrated PN group), with the exception that significantly more infants in the concentrated PN group were born after multiple pregnancy (Table 1). Unadjusted results are shown since adjusting for the different multiple pregnancy rate did not change the results.

**TABLE 1.** Perinatal and clinical outcome data during hospitalization of very low birth weight infants who received either an original or a concentrated parenteral nutrition regimen

Perinatal and outcome data	Original PN (n = 72–79)*	Concentrated PN (n = 41–46)*	*P* value†
Perinatal data	Mean ± SD	Mean ± SD	
Gestational age at birth, wk	26.7 ± 2.7	27.2 ± 2.4	0.4
Birth weight, g	898 ± 318	903 ± 306	0.9
Birth weight, SDS	−1.3 ± 1.4	−1.7 ± 1.6	0.1
	Median (range)	Median (range)	
Apgar score at five minutes	7 (2–10)	7 (2–10)	0.5‡
	n (%)	n (%)	
Sex, male	43 (54)	28 (61)	0.6
Multiple pregnancy	14 (18)	18 (39)	0.01
Antenatal steroid treatment	69 (92)	42 (95)	0.7
Small for gestational age§	20 (25)	14 (30)	0.5
Clinical outcome data	Median (range)	Median (range)	
Days of MV treatment	4 (0–60)	5 (0–59)	0.9‡
Days of antibiotics treatment	17 (0–98)	14 (0–79)	0.3‡
	n (%)	n (%)	
Postnatal steroid treatment	28 (35)	15 (33)	0.8
Treated patent ductus arteriosus∥	39 (49)	13 (28)	0.02
Necrotizing enterocolitis¶	7 (8.9)	1 (2.2)	0.3
Severe ROP (stages 3–5)#	17 (24)	8 (20)	0.8
Severe IVH (grades 3 or 4)**	7 (9.0)	6 (13.0)	0.5
Bronchopulmonary dysplasia††	25 (32)	14 (30)	1.0
Culture-verified sepsis	26 (33)	12 (26)	0.5
Early metabolic acidosis‡‡	49 (63)	24 (55)	0.4

IVH = intraventricular hemorrhage; MV = mechanical ventilation; PN = parenteral nutrition; ROP = retinopathy of prematurity; SD = standard deviation; SDS = standard deviation score.

* Different infant numbers were due to unobtainable data.

† Independent samples *t* test and the Fisher exact test were used for continuous and binary outcome variables, respectively.

‡ Mann-Whitney *U* test was used to account for the not normally distributed data.

§ Birth weight <2 SDs below the mean, using a Swedish growth reference (20).

∥ Included pharmacological and surgical intervention.

¶ Defined as stage ≥2 according to Bell et al (18).

#Classified according to the international classification of ROP (17).

** Graded according to the classification system of Papile et al (19).

†† Defined as need of oxygen or respiratory support at 36 wk postmenstrual age.

‡‡ Defined as a pH < 7.30 in combination with a base excess <–5 mmol/L, occurring at least once during postnatal days 0–6.

### Nutrient Intakes

Intakes of electrolytes, energy and amino acids were significantly higher from the concentrated PN regimen than from the original PN regimen, both during postnatal days 0 to 3 and days 4 to 6 (Table 2). In addition, total electrolyte intakes including all parenteral and enteral nutritional sources, were higher in the concentrated PN group compared with the original PN group (Figure, Supplemental Digital Content 2, http://links.lww.com/MPG/C860). Sources of parenteral nutrient intakes were (1) the respective PN regimen, (2) saline injections/infusions, and (3) blood product transfusions. The estimated contents of electrolytes in blood products are presented in Table, Supplemental Digital Content 3, http://links.lww.com/MPG/C860. During postnatal days 0 to 6, the respective PN regimen was the largest contributor to total parenteral electrolyte intakes, with a mean contribution of 56% (sodium), 54% (chloride), 98% (potassium), 99% (phosphorus), and 98% (calcium). As previously reported, the total fluid intakes were similar between the groups (7).

**TABLE 2.** Mean intakes of fluid, energy, amino acids, and electrolytes from the original and the concentrated parenteral nutrition regimen* in very low birth weight infants during the first postnatal week

Intakes	Original PN (n = 79)	Concentrated PN (n = 46)	*P* value†
Postnatal days 0–3	Mean ± SD	Mean ± SD	
Fluid, mL/kg/d	76.0 ± 14.6	73.0 ± 14.2	0.3
Energy, kcal/kg/d	41.5 ± 8.9	50.5 ± 10.1	<0.001
Amino acids, g/kg/d	1.84 ± 0.49	2.31 ± 0.44	<0.001
Sodium, mmol/kg/d	0.75 ± 0.44	2.04 ± 0.52	<0.001
Chloride, mmol/kg/d	0.54 ± 0.41	1.79 ± 0.50	<0.001
Potassium, mmol/kg/d	0.47 ± 0.21	1.16 ± 0.29	<0.001
Phosphorus, mmol/kg/d	0.46 ± 0.22	1.06 ± 0.23	<0.001
Calcium, mmol/kg/d	0.53 ± 0.16	1.00 ± 0.22	<0.001
	Ratio	Ratio	
Potassium/amino acid ratio, mmol/g	0.28	0.51	<0.001
Phosphorus/amino acid ratio, mmol/g	0.25	0.46	<0.001
Calcium/phosphorus ratio, mmol/mmol	1.15/1	0.94/1	<0.001
Postnatal days 4–6	Mean ± SD	Mean ± SD	
Fluid, mL/kg/d	82.8 ± 32.2	79.2 ± 32.1	0.5
Energy, kcal/kg/d	47.3 ± 18.5	60.2 ± 23.4	0.001
Amino acids, g/kg/d	2.03 ± 0.90	2.44 ± 0.92	0.02
Sodium, mmol/kg/d	1.77 ± 1.09	2.99 ± 1.44	<0.001
Chloride, mmol/kg/d	1.48 ± 1.00	2.52 ± 1.48	<0.001
Potassium, mmol/kg/d	0.89 ± 0.45	1.33 ± 0.61	<0.001
Phosphorus, mmol/kg/d	0.79 ± 0.44	1.33 ± 0.51	<0.001
Calcium, mmol/kg/d	0.53 ± 0.25	1.04 ± 0.48	<0.001
	Ratio	Ratio	
Potassium/amino acid ratio, mmol/g	0.46	0.57	<0.001
Phosphorus/amino acid ratio, mmol/g	0.39	0.55	<0.001
Calcium/phosphorus ratio, mmol/mmol	0.67/1	0.78/1	0.6

PN = parenteral nutrition; SD = standard deviation.

* Intakes from the main bags and additional bags, including electrolyte supplements added to those. Intakes do not include other intravenous fluids, eg, flush solutions or transfusions.

† Independent samples *t* test.

The concentrated PN regimen also resulted in higher potassium/amino acid ratios and higher phosphorus/amino acid ratios. During postnatal days 0 to 3, the calcium/phosphorus ratio was lower in the concentrated PN regimen than in the original PN regimen. Fluid intakes from the 2 PN regimen did not differ during postnatal days 0 to 3 or 4 to 6. Sodium or chloride intakes from saline injections/infusions and electrolyte intakes from blood product transfusions did not differ between the groups during the first postnatal week (Figure, Supplemental Digital Content 2, http://links.lww.com/MPG/C860).

During postnatal days 0 to 3, enteral nutrition contributed on average with 6% sodium, 11% chloride, 26% potassium, 14% phosphorus, and 17% calcium (10% when calcium intake was corrected for the enteral absorption rate (12) of total intakes) (Figure, Supplemental Digital Content 2, http://links.lww.com/MPG/C860). Enteral electrolyte intakes during days 0 to 3 were slightly higher in the concentrated PN group than in the original PN group for sodium (0.19 vs 0.16 mmol/kg/d, *P* = 0.09), chloride (0.36 vs 0.29 mmol/kg/d, *P* = 0.05), potassium (0.30 vs 0.24 mmol/kg/d, *P* = 0.02), phosphorus (0.13 vs 0.10 mmol/kg/d, *P* = 0.03), and calcium (0.17 vs 0.13 mmol/kg/d, *P* = 0.03); however, these differences were all <1/10 of the differences in parenteral intakes. During postnatal days 4 to 6, enteral intakes of sodium (0.78 vs 0.63 mmol/kg/d, *P* = 0.31), chloride (1.18 vs 0.95 mmol/kg/d, *P* = 0.17), potassium (0.84 vs 0.68 mmol/kg/d, *P* = 0.10), phosphorus (0.40 vs 0.30 mmol/kg/d, *P* = 0.13), and calcium (0.56 vs 0.42 mmol/kg/d, *P* = 0.11) did not differ significantly between the concentrated and the original PN group and contributed on average between 18% and 41% of total electrolyte intakes.

Electrolyte supplements were commonly added to the PN bags (Table, Supplemental Digital Content 4, http://links.lww.com/MPG/C860). Phosphorus and sodium intakes from sodium glycerophosphate supplements were higher in the concentrated PN group, while intakes from chloride, potassium and calcium supplements were lower compared with the original PN group.

### Electrolyte Imbalances

**FIGURE 1.**
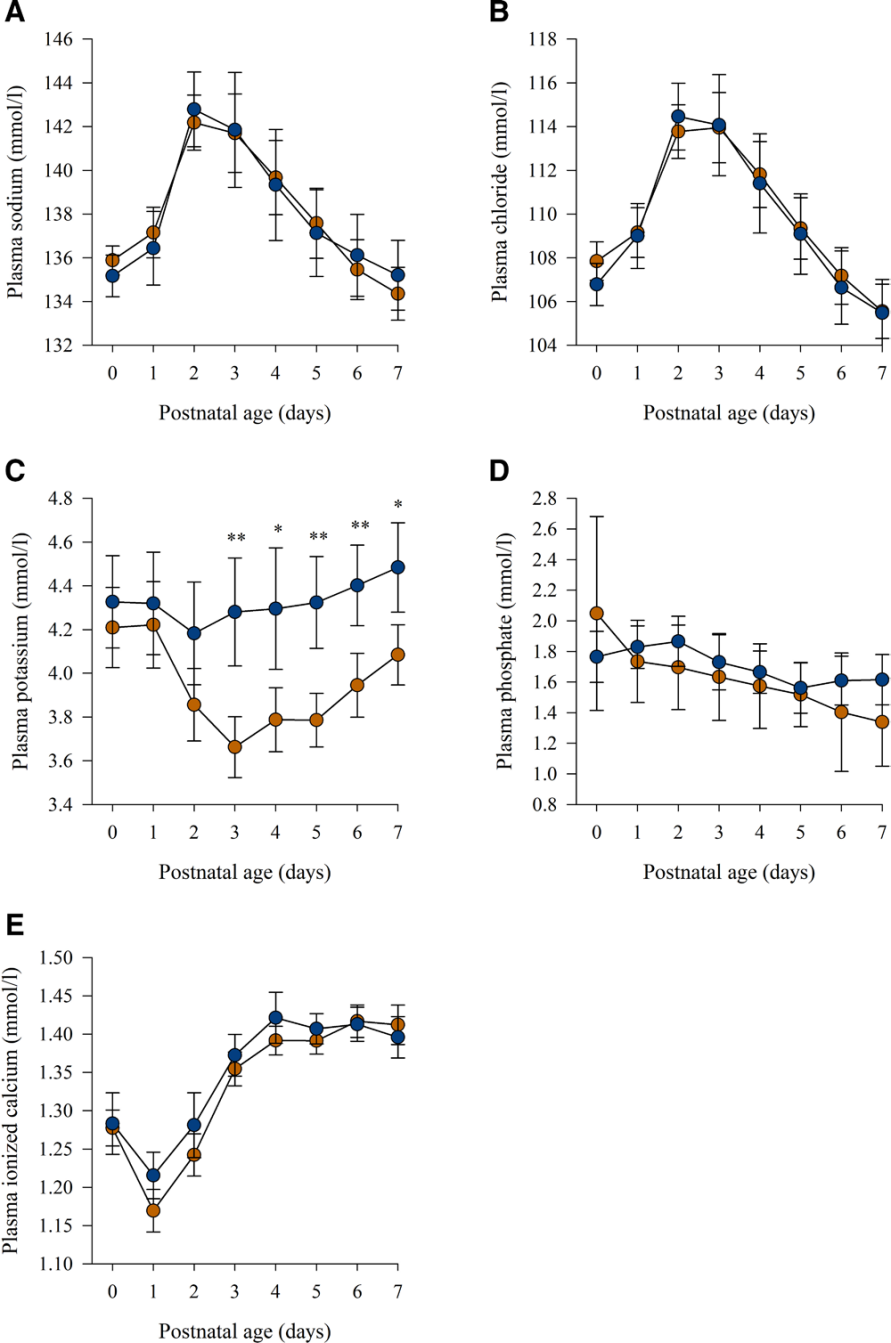
A–E, First week electrolyte concentrations of very low birth weight infants who received either an original (— ● —) or a concentrated (— ● —) parenteral nutrition regimen. Different infant numbers for sodium, chloride, potassium, ionized calcium (n = 76–79 and n = 41–46), and phosphate concentrations (n = 6–20 and n = 16–30) were due to unobtainable data. Values are expressed as 95% confidence interval for mean. Independent samples *t* test. *P < 0.01, **P < 0.001.

Plasma concentrations of sodium, chloride, phosphate, and ionized calcium and the prevalence of hypernatremia, hyperchloremia, hypochloremia, hyperkalemia, and hypercalcemia did not differ between the groups (Fig. 1A, B, D, E, Table 3). Plasma potassium concentrations were higher during postnatal days 3 to 7 in the concentrated PN group compared with the original PN group (Fig. 1C). Infants in the concentrated PN group had significantly less often hypokalemia and severe hypophosphatemia during postnatal days 0 to 7 (Table 3).

**TABLE 3.** Electrolyte imbalances during the first postnatal week in very low birth weight infants who received either an original or a concentrated PN regimen

Electrolyte imbalances*	Original PN (n = 79)	Concentrated PN (n = 46)	*P* value†
	n (%)	n (%)	
Hypernatremia, >145 mmol/L	24 (30)	16 (35)	0.7
Hyperchloremia, >120 mmol/L	16 (20)	8 (17)	0.8
Hypochloremia, <100 mmol/L	12 (15)	11 (24)	0.2
Hypokalemia, <3.5 mmol/L	60 (76)	14 (30)	<0.001
Severe hypokalemia, <3.0 mmol/L	11 (14)	3 (6.5)	0.3
Hyperkalemia, >6.0 mmol/L	8 (10)	6 (13)	0.8
Hypophosphatemia, <1.4 mmol/L	27 (45), n = 60‡	18 (40), n = 45‡	0.7
Severe hypophosphatemia, <1.0 mmol/L	10 (17), n = 60†	1 (2.2), n = 45†	0.02
Hypercalcemia, >1.45 mmol/L	35 (44)	25 (54)	0.4

PN = parenteral nutrition.

* Defined according to the definitions used by the European Society for Paediatric Gastroenterology, Hepatology and Nutrition (ESPGHAN) (8) and according to the scientific literature (4,13–16), if occurring at least once during postnatal days 0 to 7.

† Fisher exact test.

‡ Different infant numbers were due to unobtainable data.

### Clinical Outcome Data

Infants in the concentrated PN group less often received pharmacological and surgical treatment for patent ductus arteriosus compared with infants in the original PN group (Table 1). Duration of mechanical ventilation and antibiotics treatment as well as treatment with postnatal steroids did not differ between the groups. The prevalence of necrotizing enterocolitis, intraventricular hemorrhage grades 3 or 4, bronchopulmonary dysplasia, culture-verified sepsis, ROP stages 3 to 5, and early metabolic acidosis did not differ between the groups (Table 1).

## DISCUSSION

This study shows that an energy- and amino acid-optimized concentrated PN regimen with a higher content of sodium, chloride, potassium, phosphorus, and calcium does not cause increased electrolyte imbalances during the first postnatal week in VLBW infants. Indeed, fewer infants who received the concentrated PN regimen had hypokalemia and severe hypophosphatemia. Furthermore, the prevalence of early hypernatremia was not increased in infants who received concentrated PN containing more sodium.

The postnatal increase of plasma sodium is partly the result of the physiological adaptation process and the loss of extracellular water, but hypernatremia is known to be affected by the amount of sodium supplied to the infants (5,22,23). Current PN recommendations suggest a minimal sodium intake during the first days of life of 0 to 2 (3) mmol/kg/d, and it is thus commonly considered that there is a need for a special low-sodium PN solution during the first days after birth to prevent early hypernatremia. Average sodium intakes of 1.1 mmol/kg/d have been shown to be associated with a low risk of hypernatremia (4) but modern PN solutions may result in sodium intakes twice as high, mainly due a higher content of sodium glycerophosphate. In the present study, daily plasma sodium concentrations and the prevalence of hypernatremia during the first postnatal week were almost identical in the 2 groups, despite the considerably higher sodium intake of infants in the concentrated PN group. Accordingly, an initial low-sodium PN solution might not be necessary, since a PN solution providing 2 mmol/kg/d of sodium during the first 3 days of life did not increase the prevalence of hypernatremia, which is a novel finding. The results for chloride were very similar to the results for sodium.

Current recommendations suggest relatively high total energy and protein intakes, which have been associated with improved postnatal growth (7). However, higher amino acid intakes may increase the risk of hypophosphatemia and hypokalemia and thus require a higher intake of phosphate and potassium (13,24,25).

Early hypophosphatemia and hypokalemia have recently been recognized as significant problems in VLBW infants receiving currently recommended levels of parenteral amino acids without adequate provision of electrolytes. These early electrolyte disturbances are mainly caused by a shift of electrolytes between body fluid compartments (3). In a randomized controlled study by Moltu et al (13), the majority of VLBW infants who received an enhanced parenteral feeding protocol developed hypokalemia and hypophosphatemia, respectively, during the first postnatal week. Infants in our concentrated PN group received similar amounts of energy and protein as the enhanced group in the study of Moltu et al but higher amounts of potassium (1.8 vs 1.3 mmol/kg/d) and phosphorus (1.5 vs 0.5 mmol/kg/d). This higher potassium and phosphorus supply was associated with a much lower prevalence of hypokalemia (30% vs 88%) and hypophosphatemia (40% vs 77%) in our concentrated PN group compared with Moltu et al. In contrast, 76% of the infants in our original PN group had hypokalemia, receiving 1.1 mmol total potassium/kg/d during the first postnatal week. Our data confirm the results of Moltu et al that a first week total potassium intake of 1.1 to 1.3 mmol/kg/d is insufficient, when protein intake is according to the recommendations (7,13,26). Phosphorus (and sodium) intakes from sodium glycerophosphate supplements were higher in the concentrated group, likely due to the higher amino acid intake but also due to more awareness of hypophosphatemia during the latter time period. However, most of the phosphorus was provided by the PN bags in both groups. Our present data indicate that the increased supply of electrolytes by the concentrated PN regimen, including additional phosphorus supplements, provided the necessary amounts to compensate for the higher protein intake while the original PN regimen provided insufficient amounts of potassium and phosphorus in relation to protein intake.

Current PN guidelines for preterm infants recommend a potassium intake of 0 to 3 and 2 to 3 mmol/kg/d during postnatal days 1 to 3 and 4 to 5, respectively (8). During the first 3 postnatal days, infants in our concentrated PN group compared with our original PN group received more total potassium (mean intake: 1.5 vs 0.7 mmol/kg/d) and had significantly higher plasma potassium concentrations already from postnatal day 3. Therefore, an increase of the lower limit of recommended potassium intake during the first 3 postnatal days, giving a range of 1 to 3 mmol/kg/d might be useful to prevent initial hypokalemia. During postnatal days 4 to 6, the mean total potassium intake of the concentrated PN group was according to the recommendations, while the potassium intake of the original PN group was lower than recommended, confirming that a potassium intake below the recommendations may cause hypokalemia. Furthermore, considerably less potassium supplements were given in the concentrated PN group, likely due to little need of these supplements since the potassium intakes from the concentrated PN solution was higher. Based on our results and the fact that potassium requirement depends on the intake of protein or amino acids, we suggest a potassium/amino acid ratio of 0.5 to 0.6/1 (mmol/g) to avoid hypokalemia in VLBW infants during the first postnatal week.

For phosphorus, an intake of 1 to 2 mmol/kg/d and a molar calcium/phosphorus ratio of 0.8 to 1/1 during the first postnatal days is recommended (9). During postnatal days 0 to 3, infants in the original PN group did not reach the recommended phosphorus intake and the calcium/phosphorus ratio was higher than recommended, leading to a relatively high prevalence of severe hypophosphatemia. Accordingly, a study by Mulla et al showed that in preterm infants (<37 weeks gestational age) receiving amino acids ≥2.5 g/kg/d from postnatal day one a molar calcium/phosphorus ratio of 1.0/1 compared with a ratio of 1.3 to 1.5/1 was associated with a lower prevalence and severity of hypophosphatemia and hypercalcemia (27). Our present results indicate that besides a low calcium/phosphorus ratio, a relatively high phosphorus/amino acid ratio of about 0.5 (mmol/g) during the first postnatal week might prevent severe hypophosphatemia.

Strengths of the present study include the detailed and extensive data collection. Limitations are the retrospective study design and that electrolyte losses via skin, feces, and urine could not be considered. Furthermore, plasma phosphate concentrations were not measured on a daily basis in clinical routine, and thus the results are based on a lower number of measured phosphate concentrations compared with the other electrolytes. Sodium glycerophosphate supplements were given more often in the concentrated PN group, likely due to more awareness of hypophosphatemia during the latter time period, making it more difficult to interpret the results with regard to phosphorus. In addition, plasma concentrations of electrolytes do not accurately reflect total body electrolyte status and are affected not only by the electrolyte supply from PN but also by many other factors; thus, we cannot conclude with certainty that the PN provided in the concentrated PN group results in whole body sufficiency of these electrolytes. Also, the results may not be generalizable to parenteral regimens with very different fluid or electrolyte intakes.

## CONCLUSIONS

A concentrated PN regimen with relatively high intakes of sodium (2 mmol/kg/d) during the first 3 days of life was well tolerated in VLBW infants, suggesting that there might not be a need of a special low-sodium PN solution during the first postnatal days. An advantage of a higher sodium tolerance is that it allows a higher phosphorus intake from sodium glycerophosphate. Furthermore, a higher parenteral potassium intake was associated with lower risk of hypokalemia, suggesting that the minimum recommended potassium intake during the first 3 postnatal days should be increased from 0 to 1 mmol/kg/d in VLBW infants.

## Acknowledgments

We thank Maria Öström, Jennifer Ring, Mia Lindberg, Emma Edner, Stina Alm, Lena Hansson, and Itay Zamir for entering and checking nutrition and laboratory data.

## Supplementary Material


