# Supplementary material for: Higher Parenteral Electrolyte Intakes in Preterm Infants During First Week of Life: Effects on Electrolyte Imbalances
Source: J Pediatr Gastroenterol Nutr. 2022 Jun 20;75(3):e53–9. doi: 10.1097/MPG.0000000000003532 (PMC9365265; doi:10.1097/MPG.0000000000003532)
Supplement: Supplementary file 1 [file mpg-75-e53-s001.pdf]

Table, Supplemental Digital Content 1. Electrolyte and macronutrient content of the parenteral nutrition (PN) bags given to very low birth weight infants.

Electrolyte and macronutrient content in main bag[1]	Original PN	Concentrated PN
Sodium, mmol/100ml	1.05	2.7
Chloride, mmol/100ml	-	3.9
Potassium, mmol/100ml	1.05	2.6
Phosphorus, mmol/100ml	1.5[2]	1.3[3]
Calcium, mmol/100ml	0.6	1.6
Calcium/phosphorus ratio, mmol/mmol	0.4/1	1.2/1
Further electrolytes added to	Additional bag[4]	Main bag
Glucose, g/100ml	6.84	16.7
Amino acids, g/100ml	2.72	3.9
Lipids, g/100ml	1.66	0.0[5]

[1]Both main bags contained glucose, amino acids, electrolytes. The Original main bag also contained lipids and vitamins.

[2]The original main bag contained inorganic phosphorus (disodium phosphate and potassium dihydrogen phosphate).

[3]The concentrated main bag contained organic phosphorus (sodium glycerophosphate). The concentrated additional bag (lipid solution) contained an additional 1.5 mmol phosphorus/100ml.

[4]The Original additional bag contained glucose and individually added electrolytes.

[5]The Concentrated additional bag contained lipids (SMOF, i.e. Soybean oil, Medium chain triglycerides, Olive oil, and Fish oil) and vitamins.

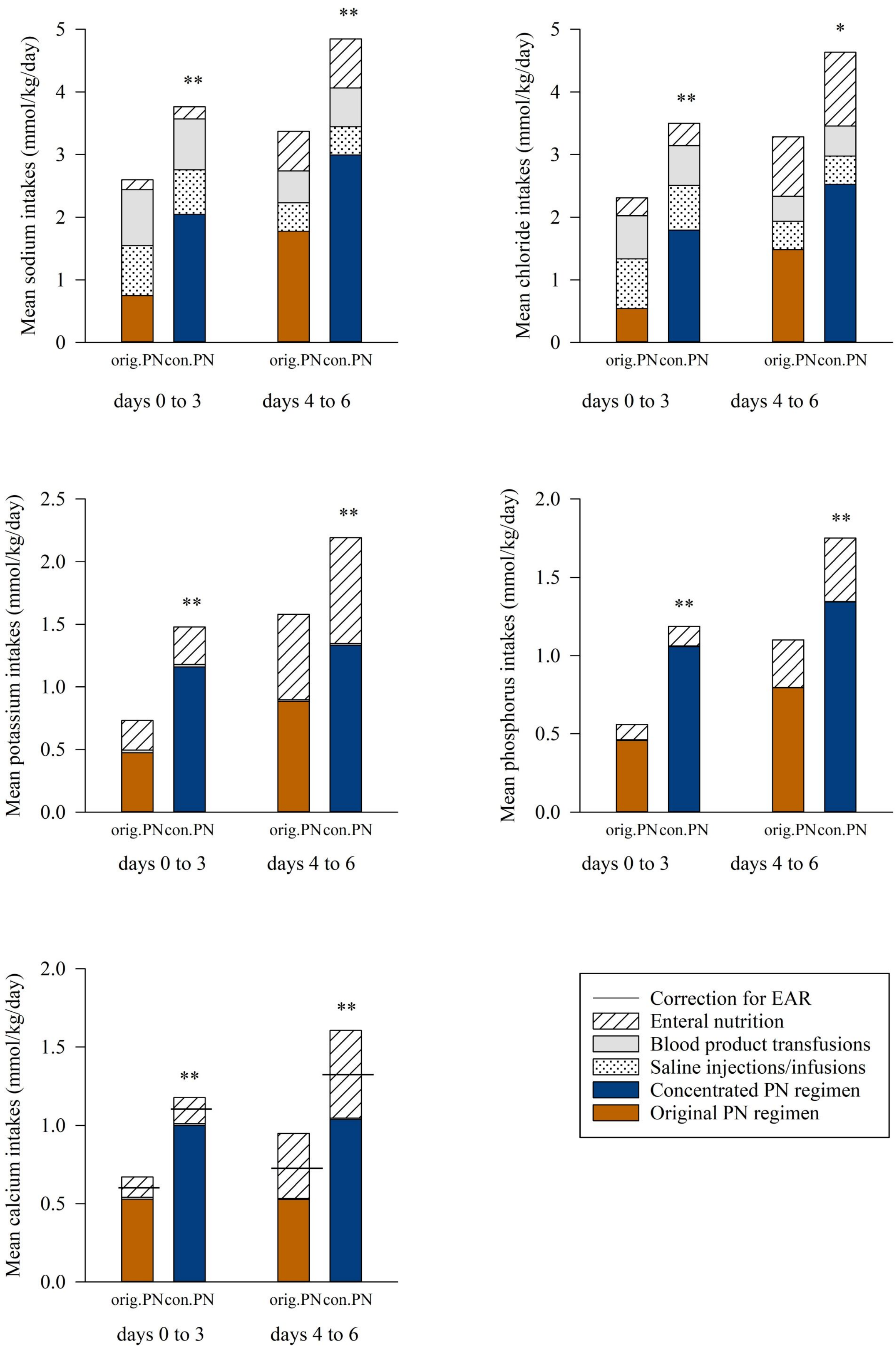

Mean sodium intakes (mmol/kg/day)
Mean chloride intakes (mmol/kg/day)
Mean potassium intakes (mmol/kg/day)
Mean phosphorus intakes (mmol/kg/day)
Mean calcium intakes (mmol/kg/day)
orig.PN
con.PN
days 0 to 3
days 4 to 6
**
*
Correction for EAR
Enteral nutrition
Blood product transfusions
Saline injections/infusions
Concentrated PN regimen
Original PN regimen

Table, Supplemental Digital Content 3. Electrolyte content in blood products given to very low birth weight infants.

Electrolyte content	Erythrocytes	Plasma
Sodium, mmol/1000ml	107	128
Chloride, mmol/1000ml	86	93
Potassium, mmol/1000ml	2.2	3.7
Phosphorus, mmol/1000ml	0.5	0.9
Calcium, mmol/1000ml	1.3	2.1

Table, Supplemental Digital Content 4. Intakes of electrolytes provided by supplements* added to parenteral nutrition bags.

Time period and Electrolyte	Original PN (n=79)	Concentrated PN (n=46)	*P*[a]
Postnatal days 0 to 3	Mean ± SD (%[b])	Mean ± SD (%)	
Sodium, mmol/kg/d	0.49 ± 0.43 (66%)	0.78 ± 0.42 (38%)	<0.001
Chloride, mmol/kg/d	0.52 ± 0.37 (97%)	0.08 ± 0.17 (5%)	<0.001
Potassium, mmol/kg/d	0.22 ± 0.17 (48%)	0.02 ± 0.05 (2%)	<0.001
Phosphorus, mmol/kg/d	0.09 ± 0.20 (20%)	0.36 ± 0.19 (34%)	<0.001
Calcium, mmol/kg/d	0.36 ± 0.15 (68%)	0.27 ± 0.12 (27%)	0.001
Postnatal days 4 to 6			
Sodium, mmol/kg/d	1.34 ± 1.04 (76%)	1.82 ± 1.21 (61%)	0.020
Chloride, mmol/kg/d	1.44 ± 0.96 (97%)	0.77 ± 1.18 (31%)	0.001
Potassium, mmol/kg/d	0.47 ± 0.39 (53%)	0.16 ± 0.28 (12%)	<0.001
Phosphorus, mmol/kg/d	0.17 ± 0.37 (21%)	0.58 ± 0.28 (44%)	<0.001
Calcium, mmol/kg/d	0.30 ± 0.21 (56%)	0.32 ± 0.23 (31%)	0.567

*The following parenteral supplements were included in the calculations: Sodium chloride, sodium glycerophosphate, potassium chloride, dipotassium phosphate, potassium hydroxide, calcium gluconate and calcium lactobionate.

[a]Independent samples t-test

[b]Percentages are proportion of total PN intake

PN, parenteral nutrition; SD, standard deviation.
